# Supplementary material for: Dynamics of hunchback translation in real-time and at single-mRNA resolution in the Drosophila embryo
Source: Development. 2021 Apr 15;148(18):dev196121. doi: 10.1242/dev.196121 (PMC8077512; doi:10.1242/dev.196121)
Supplement: Reviewer comments [file develop-148-196121_review_history.pdf]

Dynamics of *hunchback* translation in real time and at single-mRNA resolution in the *Drosophila* embryo

Daisy J. Vinter, Caroline Hoppe, Thomas G. Minchington, Catherine Sutcliffe and Hilary L. Ashe

DOI: 10.1242/dev.196121

Editor: Thomas Lecuit

Review timeline
Original submission: 18 August 2020
Editorial decision: 27 October 2020
First revision received: 18 February 2021
Accepted: 10 March 2021

Original submission

First decision letter

MS ID#: DEVELOP/2020/196121

MS TITLE: Dynamics of hunchback translation at single mRNA resolution in the Drosophila embryo

AUTHORS: Daisy J Vinter, Caroline Hoppe, and Hilary L Ashe

I sincerely apologise for the verry long delay before being able to come back to you. The currrent circumstances make the review process unusually slow at times. I have now received all the referees' reports on the above manuscript, and have reached a decision. The referees' comments are appended below, or you can access them online: please go to BenchPress and click on the 'Manuscripts with Decisions' queue in the Author Area.

As you will see, the referees express considerable interest in your work, but make useful comments and recommend a revision of your manuscript before we can consider publication. If you are able to revise the manuscript along the lines suggested, which may involve further experiments, I will be happy receive a revised version of the manuscript. Your revised paper will be re-reviewed by one or more of the original referees, and acceptance of your manuscript will depend on your addressing satisfactorily the reviewers' major concerns. Please also note that Development will normally permit only one round of major revision.

We are aware that you may be experiencing disruption to the normal running of your lab that make experimental revisions challenging. If it would be helpful, we encourage you to contact us to discuss your revision in greater detail. Please send us a point-by-point response indicating where you are able to address concerns raised (either experimentally or by changes to the text) and where you will not be able to do so within the normal timeframe of a revision. We will then provide further guidance. Please also note that we are happy to extend revision timeframes as necessary.

Please attend to all of the reviewers' comments and ensure that you clearly highlight all changes made in the revised manuscript. Please avoid using 'Tracked changes' in Word files as these are lost in PDF conversion. I should be grateful if you would also provide a point-by-point response detailing how you have dealt with the points raised by the reviewers in the 'Response to Reviewers' box. If you do not agree with any of their criticisms or suggestions please explain clearly why this is so.

© 2021. Published by The Company of Biologists under the terms of the Creative Commons Attribution License (https://creativecommons.org/licenses/by/4.0/).

Reviewer 1

Advance summary and potential significance to field

Several decades of studies have elucidated the mechanisms that establish the complex spatial expression pattern of the Drosophila gap gene hunchback (hb). This gene is expressed in three stages. First, ubiquitous hb mRNA expressed during oogenesis is translationally repressed in the posterior, creating a shallow protein gradient with highest levels in the anterior. Second, hb is activated zygotically by the Bicoid (Bcd) gradient and its cofactors in the anterior half of the embryo, which creates a sharper posterior boundary to the anterior hb domain. Third, hb is activated in two stripes that perdure after Bcd has degraded. One of those stripes, called the PS4 stripe, overlaps with the posterior boundary of the Bcd-activated hb domain, further sharpening it.

This paper shows strong visual evidence that an unknown translational mechanism is critical for down-regulating translation efficiency in regions that lie anterior to the PS4 stripe. The key reagent is a hb reporter transgene that is transcriptionally activated by Bcd, but lacks the enhancer element(s) that direct PS4 stripe expression. The hb reporter is pretty complicated, as it contains a Suntag (24 copies of a GCN4 epitope) fused to the N-terminus of the protein coding region and 24 copies of an MS2 RNA hairpin inserted into the 3'UTR. Curiously, the authors were not able to detect the MS2 hairpins. Instead, they used smFISH to show that the mRNA directed by this construct is expressed evenly throughout the anterior half of the embryo. In contrast, their Suntag detection method, which uses a newly engineered detection transgene (scFv-mNeonGreen) that contains an NLS to sequester unbound Neon Green into the nucleus, and permits the detection of individual translation events in the cytoplasm, shows that translation efficiency peaks in a broad stripe centered over the PS4 position. This is a very interesting result that lays the groundwork for future studies to identify which parts of the hb mRNA are involved in this mechanism.

Comments for the author

1. For me, the main question is whether the data obtained from this transgene reflects the activities of the endogenous gene. One possibility is that the repeated sequences reduce the stability of the mRNA, which is consistent with the failure to detect it using the MS2 system. It would be nice to see this experiment repeated with a transgene that lacks the hairpins. In addition, it would be nice to see whether the transgene, which contains the full hb coding sequence, can provide any rescue activity in a hb mutant.

2. The authors state that the reporter was inserted into two different genomic positions. Were similar results obtained with both? This should be mentioned.

3. All the data shown is from a single copy transgene in a wild-type embryo. Does the data change if two three, or four copies are examined? Showing that the quantitative image data (# of mRNAs, protein initiations etc.) correlates with copy number would strengthen the paper.

4. The description of what is known about the expression pattern of hb could be improved. Some mention of the early work that identified the mechanism of PS4 stripe-specific expression (Margolis et al., 1995 Dev) and the dynamic changes in hb expression (Wu et al., 2001 Dev Biol) should be included.

Reviewer 2

Advance summary and potential significance to field

This manuscript uses the elegant Sun tag approach and FISH to visualise nascent Hunchback protein attached to its mRNA in order to quantify how efficiently the mRNA is translated in different regions of the embryo. Their results suggest that the translation of hb mRNA is specifically repressed anteriorly during nuclear cycle 14 although the biological significance of this repression is unknown. Given the intense effort that has gone into understanding and modeling the behaviour of the gap genes, the discovery of a new step that regulates Hb protein levels will be of interest even though there is no mechanistic data.

© 2021. Published by The Company of Biologists under the terms of the Creative Commons Attribution License (https://creativecommons.org/licenses/by/4.0/).

Comments for the author

This short report uses advanced imaging techniques to examine the translation of hb mRNA in situ by visualising the nascent polypeptides at the same time as the RNA. This is the first time that this approach has been applied in a whole organism and represents an advance that may prove useful in other systems. The data seem to have been rigorously and carefully analysed and the results generally support the conclusions. The work does not go very far and is more a description of a new technique with one result to show that it can uncover new biology, and it is a shame that they made no attempt to investigate the mechanism of hb translational repression anteriorly or to test whether this is important. Nevertheless I think that this is a useful contribution and that it may be appropriate for publication in Development once the authors have addressed a few methodological issues.

1) The method used to analyse mRNA compaction is not adequately explained, leaving me unconvinced by the conclusion. As far as I can tell, they used single molecule imaging approaches to calculate the centres of the signals from the Sun Tags coding region and the hb coding region and then calculated the distance between them. My problem with this is that the two sequences are adjacent and there should be no distance between them. We are not told what probes they used for the hb coding region and whether these span the whole length of the RNA. In any case they did not label the hb3'UTR, so it is unclear to me how they rule out circularisation of the RNA through an interaction between the 5' and 3' ends.

2) The manuscript does not state what antibodies were used.

3) The mNeon Green tagged nanobody against the Sun Tag contains a nuclear localisation signal and appears to be nuclear in Figure 1B. This nuclear signal is not visible in any of the embryos that express the nanbody and sun tag hunchback, however, even when there is no translation of the Sun Tag. Can the authors explain why this is the case? Are they confident that there is an excess of the anti-Sun Tag nanobody so that it efficiently labels all of the translated Sun Tags. Why does it not accumulate in the nucleus with Hunchback protein?

First revision

<u>Author response to reviewers' comments</u>

Please note that this revised manuscript is now written in an Article format rather than as a shorter Report. As such there is a new separate Discussion section. After consultation with Laetitia Beck, Development's Editorial Administrator, it was agreed that it would be more helpful for us to only highlight changes in the text where our findings have changed (we have fixed the issue with the probes for analysing compaction, see below, and corrected our analysis of ribosome number in Fig.4) and where we have added new data. We also briefly highlight that, as requested, we have tested a new *SunTag-hb* transgene lacking MS2 loops, although our findings are the same.

Reviewer 1 Comments for the Author:
1. For me, the main question is whether the data obtained from this transgene reflects the activities of the endogenous gene. One possibility is that the repeated sequences reduce the stability of the mRNA, which is consistent with the failure to detect it using the MS2 system. It would be nice to see this experiment repeated with a transgene that lacks the hairpins.
We have now repeated the fixed embryo imaging with a *SunTag-hb* transgene that lacks any MS2 loops, which shows the same regulation of restricted mRNA translation in nc14 (Figs. 2, 3). The reason we could not detect the mRNA carrying the MS2 loops using live imaging was because we only had 24 MS2 copies inserted rather than 128xMS2, which gives the higher sensitivity needed to visualise single mRNAs (24xMS2 is traditionally used to visualise transcription sites where there are multiple copies of the MS2 loops from many Pol II transcribing the gene). We now also include data in the revised manuscript showing that we can image *SunTag-hb* mRNA translation sites in real time during early embryogenesis (Figs. 5, 6).

© 2021. Published by The Company of Biologists under the terms of the Creative Commons Attribution License (https://creativecommons.org/licenses/by/4.0/).

In addition, it would be nice to see whether the transgene, which contains the full hb coding sequence, can provide any rescue activity in a hb mutant.
We already know from another study (Perry et al, 2012, Curr Biol) that the proximal enhancer that we used does not rescue *hb* mutants, as it only drives transcription of *hb* in the anterior of the embryo (so lacks the contribution from the stripe enhancer). When the *hb* transcriptional dynamics were uncovered, representing the first live analysis of transcription in the *Drosophila* embryo, both labs used only the proximal enhancer to drive expression the *hb*-MS2 transgene (Lucas et al, 2013; Garcia et al, 2013, both Curr Biol). The rationale of our study was to use this well characterised expression system to now explore *hb* mRNA translation dynamics, albeit focusing on mRNAs transcribed in response to only one of the *hb* enhancers. Imaging translation of the full complement of *hb* mRNAs from all the regulatory regions would require CRISPR engineering of the SunTag sequence into the endogenous locus and is therefore beyond the scope of this study.

2. The authors state that the reporter was inserted into two different genomic positions. Were similar results obtained with both? This should be mentioned.
We apologise for our wording in the Methods, which has confused the reviewer. We wrote 'The attB-attP system was used for site specific reintegration of transgene into sites 25C6 (Chr2) and 86Fb (Chr3)' meaning that the ScFv-NeonGreen transgene was inserted into 25C6 and the Suntag-*hb* transgene was inserted into 86Fb. We only have the Suntag-*hb* transgene inserted into one genomic location (on the 3rd chromosome), but note that the Suntag-*hb* mRNA profile we determine across the AP axis is the same as that published by Garcia et al for endogenous *hb* mRNA, arguing against any artefact associated with the insertion site.

3. All the data shown is from a single copy transgene in a wild-type embryo. Does the data change if two, three, or four copies are examined? Showing that the quantitative image data (# of mRNAs, protein initiations, etc.) correlates with copy number would strengthen the paper.
Our data from analysis of embryos carrying 2 copies of the *SunTag-hb* transgene are consistent with the core observation of the paper, i.e. that we detect translation of *SunTag-hb* mRNAs across the expression domain at nc13 but only in a posterior band at nc14. However, comparing the data obtained from embryos carrying 2 vs 1 copy of the *SunTag-hb* transgene reveals some interesting findings. In embryos carrying 2 copies of the *SunTag-hb* transgene we detect broadly twice the mRNA in nc13 embryos at ~12-36% EL, as shown in the figure below (arrowheads highlight 2 transcription sites in the nucleus, the graph shows the mean from 3 embryos for nc14 and nc13 (one copy) and 2 embryos for nc13 (two copies), the area on the total mRNA graph at nc13 where the difference is ~ 2-fold is shaded in grey). At the posterior of the expression domain, we detect less than a 2-fold increase, consistent with limiting Bcd activator, whereas in the anterior region where Bcd levels are highest we detect a >2x increase in mRNA number. Curiously, at nc14 we detect the same number of transcripts with 1 or 2 copies of the *SunTag-hb* transgene, except at the very anterior. As we are confident in our ability to count mRNAs – our quantitation of *ush* mRNAs (a Dpp target gene) - show the predicted increase in number in response to extra Dpp (Hoppe et al, 2020, Dev Cell) and the *hb* mRNA profiles we obtain in nc12-14 are the same as published data with the primary enhancer (Garcia et al, 2013, Curr Biol; Bothma et al, 2015, eLife) – we interpret these unexpected changes as due to autoregulation at the transcriptional level. There is already evidence in the literature for Hb transcriptional autorepression and Hb binds the primary enhancer (Treisman and Desplan, 1989, Nature). Moreover, quantitative analysis of *hb* mRNA numbers in wildtype and *hb* mutant embryos suggests that autorepression starts at nc14 (Little et al, 2013, Cell). We suggest that this autorepression at the transcriptional level leads to similar *SunTag-hb* mRNA numbers in embryos carrying 1 or 2 copies of the transgene at nc14. It has also been shown for activation of endogenous *hb* that the shadow enhancer leads to repression of the primary enhancer at the most anterior region (Perry et al, 2011, PNAS). We speculate that in the absence of this mechanism, adding an extra copy of the *SunTag-hb* transgene allows a greater response to high Bcd concentration.

We have removed unpublished data provided for the reviewers in confidence.

In nc13, we detect around twice the number of translated *SunTag-hb* mRNAs on the edges of the expression domain with 2 copies of the transgene, but slightly less than this in the centre. At nc14, consistent with the overlap in the total *SunTag-hb* mRNA numbers, the number of translated mRNAs is also similar with 1 and 2 copies. As the data in our paper support translational repression when

© 2021. Published by The Company of Biologists under the terms of the Creative Commons Attribution License (https://creativecommons.org/licenses/by/4.0/).

Hb protein reaches a threshold concentration, we speculate that where the mRNA numbers are high in nc13 this repression initiates slightly earlier, dampening the number of mRNAs translated in regions of the expression domain where they are highest. Moreover, if this translational repression mediates mRNA degradation, as is common, this would contribute to the observed reduction in total mRNA numbers at nc14 with 2 copies of the transgene. In nc14, the number of translated mRNAs is very similar with either 1 or 2 copies of the transgene, consistent with the overlap in the total mRNA numbers. In the future, we plan to extend the research reported in this manuscript by exploiting the SunTag approach to simultaneously monitor both *Suntag-hb* transcriptional and translational changes in embryos with 1 or 2 copies of the *SunTag-hb* transgene and when endogenous *hb* levels are manipulated, to more fully understand the interplay between the negative regulation that appears to be functioning at both the transcriptional and translational levels.

4. The description of what is known about the expression pattern of hb could be improved. Some mention of the early work that identified the mechanism of PS4 stripe-specific expression (Margolis et al., 1995 Dev) and the dynamic changes in hb expression (Wu et al., 2001 Dev Biol) should be included.
We have now cited these and other *hb* papers in the Introduction and Discussion. In the Introduction we have added new text summarising what is known about the *hb* expression pattern. In the Discussion, we have improved the description of how our findings may relate to other aspects of *hb* regulation.

Reviewer 2 Comments for the Author:
1) The method used to analyse mRNA compaction is not adequately explained, leaving me unconvinced by the conclusion. As far as I can tell, they used single molecule imaging approaches to calculate the centres of the signals from the Sun Tags coding region and the hb coding region and then calculated the distance between them. My problem with this is that the two sequences are adjacent and there should be no distance between them. We are not told what probes they used for the hb coding region and whether these span the whole length of the RNA. In any case they did not label the hb3'UTR, so it is unclear to me how they rule out circularisation of the RNA through an interaction between the 5' and 3' ends.
We agree that the compaction experimental design could have been improved by positioning the probes in the 3'UTR. We have moved our second set of probes so they are now at the 3' end of the transcript. As the schematic in Fig. 4A shows, there is 3 kb distance between the centres of the probe sets. We believe that this positioning of probes now allows us to draw conclusions about the proximity of the 5' and 3' ends of the mRNA. In addition, the full probe sets are shown in Supplemental Table 1.

2) The manuscript does not state what antibodies were used.
We now describe the antibodies used, with their source and dilutions in the Methods section.

3) The mNeon Green tagged nanobody against the Sun Tag contains a nuclear localisation signal and appears to be nuclear in Figure 1B. This nuclear signal is not visible in any of the embryos that express the nanbody and sun tag hunchback, however, even when there is no translation of the Sun Tag. Can the authors explain why this is the case? Are they confident that there is an excess of the anti-Sun Tag nanobody so that it efficiently labels all of the translated Sun Tags. Why does it not accumulate in the nucleus with Hunchback protein?
The nuclear scFv-NG signal is visible in the posterior of embryos that also express the transgene, e.g. Figs 1C, 2A, 3A. It is less obvious in the anterior where the *SunTag-hb* transgene is expressed, because it is masked by the signal from translation sites and cytoplasmic proteins. From Movie S1 and Fig. 5, it appears that significant nuclear import of SunTag-Hb occurs during nc14. It is possible that nuclear import of SunTag-Hb is somewhat delayed due to binding of the scFv-NG (which obviously adds significant mass to the protein). In order to address whether the scFv-NG fusion protein is in excess, we made a new transgenic line that integrates it into a different chromosome. This has allowed us to visualise translation of *SunTag-hb* mRNAs in embryos from females with 3 or 4 copies of the scFv-NG transgene (Fig. S2); the results are the same as we report in embryos from females with 2 copies. We also obtain the same results when we express a scFv-NG transgene without the NLS, to increase the cytoplasmic concentration (Fig. S2). Therefore, we are confident that the scFv-NG protein is in excess.

© 2021. Published by The Company of Biologists under the terms of the Creative Commons Attribution License (https://creativecommons.org/licenses/by/4.0/).

Second decision letter

MS ID#: DEVELOP/2020/196121

MS TITLE: Dynamics of hunchback translation in real time and at single mRNA resolution in the Drosophila embryo

AUTHORS: Daisy J Vinter, Caroline Hoppe, Thomas G Minchington, Catherine Sutcliffe, and Hilary L Ashe
ARTICLE TYPE: Research Article

I am happy to tell you that your manuscript has been accepted for publication in Development, pending our standard ethics checks.

Reviewer 1

Advance summary and potential significance to field

Please see previous review.

Comments for the author

The authors have responded well to my comments and suggestions. In particular, repeating the experiment without a construct that lacks the MS2 hairpins strengthens the main conclusions of the paper. Also the discussion of the results of comparing embryos carrying different numbers of transgene copies is a major plus. I now support publishing this novel and interesting work.

Reviewer 2

Advance summary and potential significance to field

As before

Comments for the author

The authors have done an excellent job in responding to both referees comments and I am now happy to recommend that the manuscript be accepted in its current form.

© 2021. Published by The Company of Biologists under the terms of the Creative Commons Attribution License (https://creativecommons.org/licenses/by/4.0/).
